# Efficacy of a Digital Peer Support Program on Weight Management and Mental Health in University Students With Preobesity: Randomized Controlled Trial

**DOI:** 10.2196/78960

**Published:** 2026-01-21

**Authors:** Xingyu Liu, Ting Liu, Tao Chen, Ruisi Ma

**Affiliations:** 1 Badminton Technical and Tactical Analysis and Diagnostic Laboratory, National Academy of Badminton, Guangzhou Sport University, Guangzhou, China; 2 School of Nursing, Sun Yat-sen University, Guangzhou, China; 3 School of Physical Education, Jinan University, No. 601 Huangpu Avenue West, Tianhe District, Guangzhou, Guangdong, 510632, China, 86 13828466636; 4 Key Laboratory of Guangdong Province, Guangzhou, China

**Keywords:** digital peer support, weight management, mental health, preobese, university students, randomized controlled trial

## Abstract

**Background:**

Approximately one-third of university students are overweight or obese, and a similar proportion experience anxiety or depression. Despite the interrelated nature of weight and mental health, interventions rarely address these issues simultaneously in young adults. Digital peer support interventions have the potential to promote healthy lifestyle and mental well-being. However, evidence is limited on whether a digital peer-driven approach can concurrently improve weight management and mental health in university populations with preobesity.

**Objective:**

This randomized controlled trial (RCT) evaluated the efficacy of a digital peer support program in concurrently improving weight management and mental health outcomes among university students with preobesity.

**Methods:**

In a single-blind parallel group RCT, 216 students with preobesity were allocated equally among three 6-month arms, which were a peer support intervention, an active wellness control, and a waitlist control. The peer support arm began with an interactive online workshop followed by moderated WeChat (Tencent) group discussions, daily micro tasks, biweekly group challenges, and digital badges to reinforce engagement. The active control group received the same schedule and formats but focused on general wellness topics. The waitlist group completed the same assessments without any intervention during the study period. The primary outcome measured the change in BMI from baseline to 6 months. Secondary outcomes included weekly physical activity measured in metabolic equivalent of task minutes, self-esteem, loneliness, anxiety, and depression assessed at 0, 2, 4, and 6 months. Analyses used linear mixed effects models.

**Results:**

Retention exceeded 90%. At 6 months, the peer-support group achieved a greater BMI reduction than the active control by 0.47 (95% CI −0.89 to −0.04) kg/m² and waitlist by 0.54 (95% CI −0.85 to −0.01) kg/m². Weekly metabolic equivalent of task-minutes was 129.5 higher than active control (95% CI 53.3-205.6) and 152.9 higher than waitlist (95% CI 68.4-237.4). Self-esteem increased by 1.81 points versus active control (95% CI 0.22-3.39) and 1.99 points versus waitlist (95% CI 0.21-3.76). Loneliness scores fell by 3.79 points relative to active control (95% CI −7.03 to −0.56) and by 5.02 points relative to waitlist (95% CI −8.38 to −1.66). No significant differences emerged for anxiety or depression.

**Conclusions:**

A comprehensive digital peer-support program delivered via WeChat produced modest but clinically meaningful improvements in weight management, physical activity, self-esteem, and social connectedness among undergraduates with preobesity compared with wellness control and no intervention. These findings suggest that integrating peer support into scalable digital platforms can simultaneously address physical and psychosocial health in at-risk university populations.

## Introduction

Young adults with preobesity are defined by a BMI between 25 and 29.9 kg/m² [1]. This group represents a critical yet underaddressed population in the fight against obesity [2]. University students with preobesity have a high prevalence of excess weight, with studies reporting that roughly 20%‐30% of students fall into the overweight category, with an additional 5%‐10% already meeting obesity criteria [3,4]. Unlike individuals of normal weight, these students are already on the threshold of obesity, experiencing early weight gain–related health risks and often subtle metabolic changes [5]. Crucially, even at this preobese stage, individuals already experience adverse consequences, including heightened stress, increased depressive symptoms, and lower academic performance [6-8]. This underscores that focusing on students with preobesity is not merely a preventative measure but a response to an emerging health and psychosocial burden that distinctly affects this group.

Intervening during the preobese stage in emerging adulthood may offer a pivotal window to alter lifelong health trajectories [9]. The university years are a well-recognized turning point for weight gain and lifestyle habits, commonly known as the “freshman weight gain” phenomenon. Grounded in the socioecological model, first-year transitions expose students to interpersonal, organizational, and environmental cues that favor weight gain [10,11]. The prevention of further weight gain and the promotion of modest weight loss are widely viewed as more cost-effective and achievable than treating established obesity later on [12,13]. Overweight students generally have fewer entrenched behaviors and less severe physiological impairment than individuals with obesity, meaning early intervention can capitalize on the reversibility of risk [14]. From a public health perspective, preventing the progression from overweight to obesity avoids the need for intensive medical treatments and reduces long-term health care costs [12,15,16]. Focusing on college students with preobesity offers a critical window for high-impact preventive measures, potentially delaying cardiometabolic diseases and mental health decline before they become more difficult and expensive to manage [17,18].

Despite the clear rationale for early intervention, university students with preobesity face unique challenges that current interventions rarely address adequately [8,19]. This transitional life stage features new freedoms and psychosocial pressures. Academic stress, changing peer networks, and the shift from adolescence to adulthood can negatively affect eating, activity, and mental well-being [20]. Overweight students may struggle with body image concerns and social stigma while also encountering environments filled with unhealthy food options and sedentary habits [21]. These psychosocial stressors and environmental factors intertwine, often creating a vicious cycle where weight gain and mental health issues reinforce each other [22]. However, most existing health programs operate in isolation. Weight management initiatives in college settings tend to focus solely on diet and exercise [18], whereas campus mental health services rarely integrate lifestyle change for physical health [23]. There is a notable gap in interventions that treat physical and mental health as interconnected targets. In fact, a recent review highlighted that very few programs for university students have successfully improved both health behaviors, such as diet, physical activity, and weight-related outcomes, and mental health outcomes at the same time [8]. This lack of comprehensive approaches means students with preobesity at a crucial phase often do not receive support that addresses the full range of their needs, namely managing weight while concurrently bolstering psychological resilience and well-being. Moreover, no prior study has included both an attention-matched active control and a waitlist control arm to isolate specific peer-support benefits from general engagement effects.

Digital peer-support interventions, encompassing online group chats, moderated peer discussions, peer challenges, and digital badge systems, offer a promising and innovative solution to fill this gap for young adults with preobesity [24]. According to social cognitive theory, observing peers’ successes and receiving encouragement enhances self-efficacy and outcome expectancies, thereby improving motivation, adherence, and outcomes in weight management [25]. In line with this theory, social support and positive peer influences operate through observational learning and modeling to normalize healthy behaviors and reduce the isolation or stigma an overweight student might feel, especially among university students who are highly receptive to peer influences [25,26]. By delivering such peer support via digital platforms, we can amplify its reach and convenience. Technology-based interventions are inherently adaptable, engaging students in real time, and digital platforms offer broad appeal and extensive reach across this population [27]. Through social media groups, mobile apps, or online forums, students can connect with “virtual” peers to share progress, setbacks, and encouragement [25]. Prior studies indicate that online peer communities can recreate the sense of camaraderie found in in-person support groups, providing accountability and empathy among participants with similar goals [25]. Moreover, digital peer support can be accessed on-demand and flexibly integrated into students’ busy schedules, overcoming common barriers to participation such as time constraints or embarrassment in face-to-face settings [28]. Although this approach has been previously studied, existing trials seldom focus on the critical preobese life stage, compare multiple digital support modalities within the same randomized controlled trial (RCT), or include long-term follow-up and an expanded set of psychosocial outcomes.

In light of the above, this study aimed to evaluate the efficacy of a digital peer-support program on weight management and mental health in university students with preobesity. To test this, we conducted a single-blind, parallel-group, 3-arm RCT comparing a digital peer-support intervention, an attention-matched active control, and a waitlist control, with prespecified pairwise comparisons. We hypothesized that participants in the peer-support arm would demonstrate greater improvements in BMI and mental health measures than those in the active control arm, and that both intervention arms would outperform the waitlist control. By examining this novel intervention, our goal is to address an important gap in student health promotion and contribute evidence on the integration of digital peer support in weight management and mental health prevention efforts. Our intervention was grounded in social cognitive theory and the socioecological model [29,30]. We anticipated that peer modeling within a supportive digital environment would enhance self-efficacy and motivation for healthy behaviors and provide a multilevel context for behavior change.

## Methods

### Study Design

We conducted a single-blind, parallel-group, superiority RCT entirely online over a 6-month intervention period to evaluate a brief digital peer-support intervention for weight management and mental health in university students with preobesity. The participants were drawn from 6 universities in Guangzhou, including Sun Yat-sen University, South China Normal University, Jinan University, South China Agricultural University, South China University of Technology, and Guangdong University of Foreign Studies. Participants were recruited between September 1 and October 31, 2024, and follow-up assessments were conducted at 2 months in November 2024, 4 months in January 2025, and at the study end on April 30, 2025. The study is reported in accordance with CONSORT (Consolidated Standards of Reporting Trials) guidelines and was registered prior to enrollment (ClinicalTrials.gov ID NCT06966661). Participants were recruited from university campuses via online advertisements, email bulletins, and social media postings. Interested individuals accessed a screening survey by clicking a link or QR code in the advertisement.

### Participants and Recruitment

Eligible participants were currently enrolled university undergraduate students, aged 18 years or older, with preobese weight status (defined as BMI between 25.0 and 29.9 kg/m² based on self-reported height and weight at screening). Additional inclusion criteria were access to a stable internet connection and a device for video conferencing, to enable participation in the online program. Participants were excluded if they had a BMI in the obese range (≥30 kg/m^2^) or normal range (<25 kg/m^2^) at screening, had any diagnosed medical or psychiatric condition that would interfere with study participation or require alternate care (eg, uncontrolled diabetes, an eating disorder, psychosis, or acute suicidal ideation), or were already enrolled in a weight management program or receiving psychological counseling. To ensure safety, individuals with severe depression or high acute mental health risk were directed to clinical services and not enrolled. Pregnant students were also excluded due to potential weight changes unrelated to the intervention.

### Randomization and Blinding

Eligible consenting participants were randomly assigned in a 1:1:1 ratio to 3 groups that were the digital peer support intervention group, the active control group receiving standard online wellness content, and the waitlist control group. Randomization was carried out using a computer-generated sequence. This sequence applied permuted blocks of varying sizes (6 and 9). The allocation list was prepared by an independent statistician who was not involved in participant recruitment or intervention delivery. To maintain allocation concealment, the randomization list was stored in a password-protected file on a secure server accessible only to the statistician. Study coordinators obtained the next assignment code via a secure messaging system only after participants completed baseline assessments and eligibility confirmation, which prevented recruiters from foreseeing upcoming assignments. Because the trial was conducted entirely online, sealed envelopes were not used. Instead, participants were assigned by the statistician through a secure web-based platform. The study used a single-blind design, and participants were informed that the trial compared 3 different online wellness programs but were not told which program was the primary intervention. Both the intervention and active control groups received online content and peer interaction for 6 months to control for expectation and exposure time; the waitlist control group did not receive any intervention activities during this period.

Facilitators and research staff who delivered the intervention could not be blinded due to the nature of the program. However, they were not involved in outcome assessments, and distinct facilitators managed each group without evaluative responsibilities. Follow-up data on BMI, self-esteem, loneliness, and other outcomes were collected via self-administered online surveys distributed by research assistants who were blind to group assignment and used anonymous study codes to track responses. These assistants did not have access to the randomization list. Data analysts remained blinded to group assignments until the primary analyses were completed.

### Intervention Group

Participants in this arm began with a 15-minute live interactive workshop delivered synchronously via an online videoconferencing platform. All participants joined remotely from their own devices. A trained health educator used a shared digital whiteboard and chat functions to guide the group through evidence-based guidance on balanced eating, regular movement, stress management, and resilience building using real-time polls and interactive infographics to illustrate key points in a neutral, student-friendly language while participants added their own reflections and questions. Immediately afterward, teams of 4 to 6 students convened in a dedicated WeChat group for a 1-hour conversation combining text and voice interactions under the guidance of a trained moderator, with participants sharing brief reflections on personal challenges, exploring sustainable behavior change strategies, and agreeing on 1 or 2 small health goals for the coming week while the moderator used open-ended prompts to ensure inclusive and supportive dialogue. Over the 6-month follow-up, the WeChat group delivered daily micro tasks 7 days a week, such as minute-long nutrition quizzes, morning breathing exercises, and simple step count challenges. Each completed task earned participants a digital badge displayed in the group, and automated motivational messages reinforced progress and reminded students of their goals. Every 2 weeks, the moderator launched a friendly group challenge via the WeChat group, such as collectively walking 10,000 steps in a day to foster camaraderie and mutual accountability, and the WeChat group remained open for ongoing peer support with study staff overseeing the conversation to keep discussions respectful and on topic.

#### Active Control Group

Participants followed the same schedule and digital formats, including a 15-minute workshop, a 1-hour WeChat discussion, daily micro tasks, and 2-week challenges, but the content focused on general student wellness topics, such as effective study habits, time management, and healthy sleep. WeChat discussions addressed academic strategies, identification of barriers, and the setting of weekly improvement goals.

#### Waitlist Control Group

Participants in this arm completed all baseline and follow-up assessments on the same schedule as the other 2 groups but did not receive any workshop activity, WeChat discussions, or micro task prompts during the 6-month period. They were informed that they would gain access to the full peer-support program after the final data collection. Throughout the study, they continued their usual routines without additional contact beyond survey reminders and, at the conclusion of the follow-up, they were offered the opportunity to join the interactive workshop, the WeChat peer discussions, and to receive the series of daily micro tasks.

### Procedures

After providing informed consent, each participant completed an online baseline survey capturing demographic information, self-reported height and weight, and all outcome measures. Participants were then randomized in equal proportions to 1 of the 3 arms and received secure instructions for scheduling their workshop and WeChat group times or confirmation of their waitlist status. Sessions were arranged to match participants’ availability, and reminders were sent by email and text 1 to 2 days before each workshop or discussion session. Attendance and engagement were logged by facilitators, and all WeChat group activities were monitored by study staff to document participation. Completion of the daily micro-learning tasks and the awarding of digital badges were automatically recorded via backend logs in the WeChat mini-program. Each week, the data manager generated adherence reports to flag participants who completed fewer than half of the assigned tasks or who had not earned a badge in 7 consecutive days. These participants were contacted by study staff to encourage reengagement. No participants were excluded or reassigned on the basis of missed tasks. Adherence metrics were summarized descriptively and considered in exploratory analyses but did not influence eligibility or weighting in the primary available-case analyses. Follow-up assessments were delivered online at 2 months, 4 months, and 6 months after baseline using identical survey links to maintain blinding. Nonresponders received reminders at 5 and 12 days postinvitation and a final contact attempt by phone or messaging app if needed. Participants who completed each survey received a modest gift card honorarium and, after the final follow-up, the waitlist control group was provided access to the peer support program while the study was closed to further interventions.

### Outcomes and Measures

The primary outcome was BMI, which was recorded from baseline to the 6-month assessment, with interim follow-up assessments at 2 months and at 4 months. Participants were instructed to weigh themselves in the morning before eating and to measure height against a flat wall, noting any deviations in the procedure.

Secondary outcomes encompassed changes in physical activity, anxiety, depression, mental well-being, perceived stress, general well-being, self-esteem, loneliness, and social connectedness. Physical activity was captured by the International Physical Activity Questionnaire, a 7-item measure recording the frequency and duration of walking, moderate-intensity, and vigorous activities over the previous 7 days [31]. Anxiety symptoms were assessed using the 7-item Generalized Anxiety Disorder (GAD) scale, which rates symptoms over the past 2 weeks from 0 (not at all) to 3 (nearly every day) for a total score of 0-21 [32]; the Chinese version demonstrates strong reliability, validity, and measurement invariance in university samples [33]. Depressive symptoms were measured by the 9-item Patient Health Questionnaire (PHQ), scored 0‐3 per item for a total of 0-27 [34]; Chinese validation studies report high internal consistency and test-retest reliability [35]. Mental well-being was captured by the 14-item Warwick-Edinburgh Mental Well-being Scale (WEMWBS), with each item scored 1‐5 for a total range of 14-70 [36]; the Chinese WEMWBS shows robust internal consistency and construct validity among nursing trainees and general students [37]. Perceived stress was assessed via the 10-item Perceived Stress Scale (PSS), rated 0 (never) to 4 (very often), yielding scores from 0 to 40 [38]; the Simplified Chinese version exhibits high reliability (α>.90) and a clear 2-factor structure [39]. General well-being was measured by the 5-item World Health Organization Well-Being Index (WHO-5), scored 0 (at no time) to 5 (all of the time) and converted to a 0‐100 index [40]; the Chinese WHO-5 has demonstrated good internal consistency (α≈.85) and unidimensional fit in student and patient samples [41]. Self-esteem was measured by the 10-item Rosenberg Self-Esteem Scale on a 4-point agreement scale [42]; Chinese adaptations confirm robust factorial and criterion validity [43]. Loneliness was assessed by the 20-item University of California, Los Angeles (UCLA) Loneliness Scale Version 3, with items rated 1 (never) to 4 (often) [44]; recent validation of Chinese variants supports strong reliability and validity in adolescent and university populations [45]. Social connectedness was measured by the 8-item Social Connectedness Scale (SCS), scored 1 (strongly disagree) to 6 (strongly agree) [46]; the Chinese translation demonstrates satisfactory psychometric properties in student samples [47].

### Statistical Analysis

Effect size assumptions (Cohen *d* ≈ 0.50) were derived from published digital weight management trials [48]. A priori power analysis using G*Power (Heinrich-Heine-Universität Düsseldorf) for a 3-arm, 4 timepoint repeated measures design [49], with an assumed within-subject correlation of 0.5, 2-tailed *α*=.05, and 80% power, indicated a minimum sample of 159 participants (n=53 per arm). Allowing for up to 20% attrition, we therefore enrolled 216 participants (n=72 per arm), ensuring robust power to detect the expected effects.

Primary and secondary outcomes were measured at baseline and 2, 4, and 6 months. Analyses were performed on an available-case basis. Participants who completed at least 1 follow-up assessment were included in the analysis. Missing data were less than 5% and under the missing at random assumption, linear mixed effects models estimated by maximum likelihood were used without additional imputation [50]. Each model specified fixed effects for study arm time and the study arm by time interaction and adjusted for the baseline value of the outcome. A random intercept for each participant accounted for within-subject correlation across repeated measures. The primary test of efficacy was the study arm by time interaction for BMI, and significance for this test was evaluated at 2-tailed *P* <.05. Secondary outcomes were analyzed in the same way, and false discovery rate–adjusted *P* values were reported to provide context for the full set of psychosocial measures. Model diagnostics included inspection of residuals for normal distribution and equal variance. All analyses were performed in R (version 4.2.2; R Foundation for Statistical Computing) with the *lme4* and *mice* packages, and results are presented alongside 95 % CIs [51].

### Ethical Considerations

The study protocol was reviewed and approved by the Institutional Review Board of Guangzhou Sport University (approval number 2025LCLL-057), ensuring that the research adhered to institutional and national ethical guidelines for research involving human participants. All participants provided informed consent electronically after a one-on-one discussion with a research staff member about the study procedures and eligibility criteria. Participation was voluntary, and they were informed that they could withdraw from the study at any time without consequences. Data were collected via secure online surveys and stored on a password-protected server accessible only to the research team, and personal identifiers were removed before analysis so that only deidentified, aggregated data were used for publication to protect participant confidentiality. Participants did not receive monetary or material compensation, and digital badges and peer support interactions provided within the intervention were part of the program design and served as motivational tools rather than compensation.

## Results

**Figure 1.**
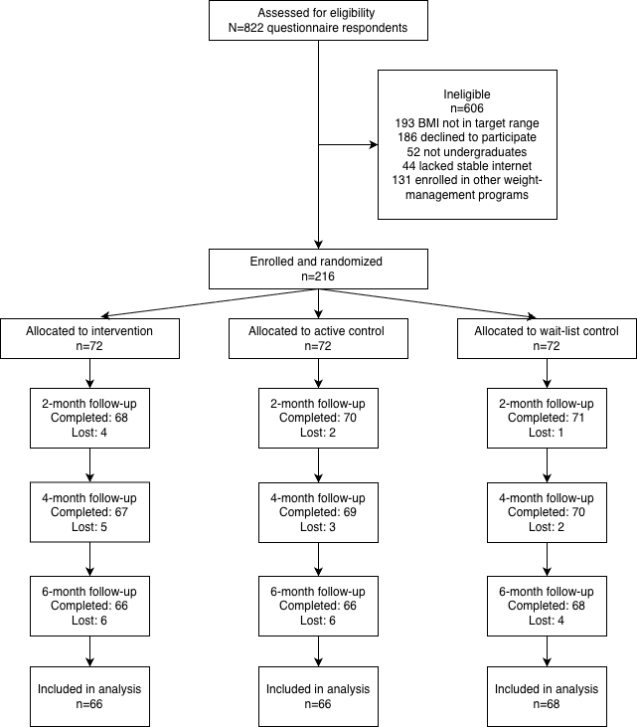
Flow diagram of participant recruitment, allocation, follow-up, and analysis in the randomized trial.

Of the 822 students assessed for eligibility, 216 met inclusion criteria and were randomized equally to the intervention (n=72), active control (n=72), and waitlist control (n=72) arms. Follow-up rates exceeded 90% at each of the 4 assessment points (Figure 1). Baseline demographic and clinical characteristics were well-balanced across groups: the overall mean age was 21.69 (SD 2.40) years, 48.15% (104/216) were female, and the mean BMI was 27.03 (SD 1.29) kg/m²; no between-group differences reached statistical significance on any measure (Table 1).

Adherence to the program activities was high. Participants in the digital peer-support arm completed an average of 130.94 out of 181 of assigned daily micro tasks (72.34%, SD 12.38%) and attended a mean of 21.68 out of 24 scheduled WeChat group discussions (90.33%). In the active wellness control arm, participants completed a mean of 124.80 out of 181 daily tasks (68.94%, SD 13.11%) and attended 21.30 out of 24 group discussions (88.73%). These adherence estimates were obtained through daily check-ins conducted by research assistants within the WeChat groups, who recorded participant confirmations of task completion and discussion attendance in study logs rather than relying on automated device logs.

**Figure 2.**
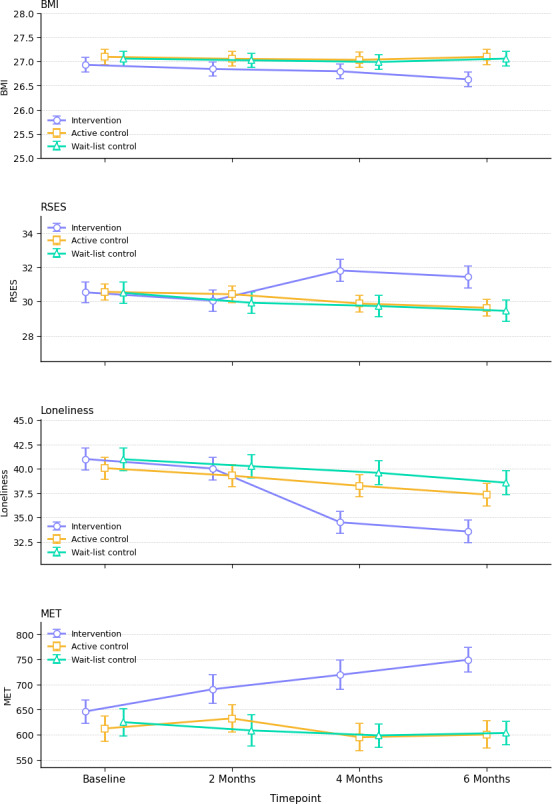
Line charts of outcomes exhibiting significant changes over time in the intervention, active control, and waitlist control groups (mean, SE). MET: metabolic equivalent of task; RSES: Rosenberg Self-Esteem Scale.

At 2 months, there were no significant group differences on any outcome. However, by 4 months (Table 2), the intervention arm had already achieved markedly higher weekly physical activity than both control groups: adjusted total metabolic equivalent of task (MET)‐minutes were 124.10 MET greater than in the active control (95% CI 45.64-202.55; *P*<.001) and 147.32 MET greater than in the wait‐list control (95% CI 62.51-232.13; *P*<.001). Self‐esteem scores in the intervention arm exceeded those of the active control by 1.93 points (95% CI 0.35-3.52; *P*=.04) and those of the wait‐list control by 2.08 points (95% CI 0.30-3.87; *P*=.04). Loneliness scores were also significantly lower among intervention participants, with scores 3.76 points below the active control (95% CI –6.93 to –.58; *P*=.03) and 5.09 points below the waitlist control (95% CI –8.40 to –1.78; *P*=.03), reflecting a greater reduction in perceived social isolation (Figure 2).

**Table 1.** Baseline demographic and psychosocial characteristics of participants, overall and by group.

Characteristics^a^	Total sample (N=216)	Intervention (n=72)	Active control (n=72)	Waitlist control (n=72)
Age, mean (SD)	21.69 (2.40)	21.83 (2.25)	21.21 (2.35)	22.04 (2.55)
Sex, n (%)				
Male	112 (51.90)	36 (50)	40 (55.56)	36 (50)
Female	104 (48.10)	36 (50)	32 (44.44)	36 (50)
Grade^b^, n (%)				
Grade 1	60 (27.78)	20 (27.78)	15 (20.83)	25 (34.72)
Grade 2	44 (20.37)	15 (20.83)	16 (22.22)	13 (18.06)
Grade 3	59 (27.31)	23 (31.94)	19 (26.39)	17 (23.61)
Grade 4	53 (24.54)	14 (19.44)	22 (30.56)	17 (23.61)
WeChat^c^ use (hrs/day), mean (SD)	1.96 (1.02)	2.04 (0.93)	2.03 (1.12)	1.82 (1)
BMI, kg/m^2^, mean (SD)	27.03 (1.29)	26.93 (1.28)	27.10 (1.34)	27.06 (1.27)
MET^d^, mean (SD)	628 (213.17)	646.40 (196.73)	612.44 (212.90)	625.14 (230.17)
GAD^e^, mean (SD)	5.31 (3.62)	5.11 (3.69)	5.79 (3.78)	5.03 (3.38)
PHQ^f^, mean (SD)	5.90 (4.53)	5.97 (4.53)	5.96 (5.04)	5.78 (4.05)
WEMWBS^g^, mean (SD)	50.30 (9.45)	49.72 (8.77)	50.19 (9.75)	50.99 (9.88)
PSS^h^, mean (SD)	16.83 (5.79)	16.97 (6.10)	17.25 (5.79)	16.28 (5.53)
WHO-5^i^, mean (SD)	61.33 (20.15)	60.67 (19.77)	64.15 (19.63)	59.17 (20.99)
Self-esteem^j^, mean (SD)	30.54 (4.84)	30.54 (5.13)	30.57 (4.06)	30.51 (5.30)
Loneliness^k^, mean (SD)	40.68 (9.62)	41 (9.71)	40.07 (9.56)	40.97 (9.68)

a Values are presented as mean (SD) for continuous variables and n (%) for categorical variables.

b Grade denotes year of study.

c WeChat use reflects average daily hours on the platform.

d MET: metabolic equivalent task minutes per week, which was assessed via the International Physical Activity Questionnaire.

e GAD: Generalized Anxiety Disorder-7.

f PHQ: Patient Health Questionnaire-9.

g WEMWBS: Warwick-Edinburgh Mental Well-being Scale.

h PSS: Perceived Stress Scale.

i WHO‐5: 5-item World Health Organization Well‐Being Index.

j Self‐Esteem: Rosenberg Self‐Esteem Scale.

k Loneliness: University of California, Los Angeles Loneliness Scale.

**Table 2.** Adjusted linear mixed‐effects model estimates with group× time interaction and pairwise contrasts.

Outcomes and timepoint^a^		Intervention	Active control	Waitlist control	Int (LRT)^b^, *χ*² (df)	Intervention versus AC^c^, mean difference (95% CI)	Intervention versus WLC^d^, mean difference (95% CI)
BMI, kg/m²							
Baseline		26.93 (1.28)	27.10 (1.34)	27.06 (1.27)	51.57 (6)	–0.17 (–0.59 to 0.26)	–0.13 (–0.55 to 0.29)
2 months		26.84 (1.27)	27.06 (1.32)	27.02 (1.28)	—^e^	–0.21 (–0.63 to 0.21)	–0.18 (–0.60 to 0.24)
4 months		26.80 (1.25)	27.03 (1.37)	26.99 (1.32)	—	–0.24 (–0.66 to 0.18)	–0.19 (–0.61 to 0.23)
6 months		26.63 (1.28)	27.10 (1.34)	27.06 (1.27)	—	–0.47 (–0.89 to –0.04)	–0.54 (–0.85 to –0.01)
MET^f^							
Baseline		646.40 (196.73)	612.44 (212.90)	625.14 (230.17)	21 (6)	33.96 (−33.11 to 101.03)	21.26 (−48.80 to 91.32)
2 months		690.64 (243.11)	632.54 (228.78)	608.71 (268.26)	—	58.10 (−19.15 to 135.34)	81.93 (−1.84 to 165.70)
4 months		719.26 (247.92)	595.17 (231.28)	598.81 (196.49)	—	124.10 (45.64-202.55)	147.32 (62.51-232.13)
6 months		749.21 (205.77)	600.42 (231.22)	603.60 (196.60)	—	129.45 (53.28-205.61)	152.87 (68.35-237.38)
GAD^g^							
Baseline		5.11 (3.69)	5.79 (3.78)	5.03 (3.38)	13.04 (6)	–0.68 (–1.83 to 0.47)	0.08 (–1.07 to 1.24)
2 months		4.67 (3.63)	5.15 (3.84)	4.38 (3.40)	—	–0.49 (–1.64 to 0.67)	0.29 (–.86 to 1.45)
4 months		4.24 (3.55)	4.75 (3.73)	3.72 (3.09)	—	–0.51 (–1.67 to 0.64)	0.51 (–0.64 to 1.67)
6 months		3.79 (3.67)	4.38 (3.78)	3.06 (3.06)	—	–0.58 (–1.74 to 0.57)	0.74 (–0.42 to 1.89)
PHQ^h^							
Baseline		5.97 (4.53)	5.96 (5.04)	5.78 (4.05)	6.33 (6)	0.01 (–1.31 to 1.33)	–0.08 (–1.40 to 1.25)
2 months		5.49 (4.33)	5.40 (4.80)	5.25 (4.02)	—	–0.49 (–1.81 to 0.83)	–0.42 (–1.74 to 0.90)
4 months		4.78 (4.41)	4.85 (4.81)	4.71 (3.87)	—	–0.51 (–1.83 to 0.81)	–0.74 (–2.06 to 0.57)
6 months		4.12 (4.33)	4.46 (4.62)	3.83 (3.70)	—	–0.51 (–1.83 to 0.81)	–0.65 (–1.97 to 0.67)
WEMWBS^i^							
Baseline		49.72 (8.77)	50.19 (9.75)	50.99 (9.88)	7.21 (6)	–0.47 (–4 to 3.06)	–2.67 (–6.20 to 0.87)
2 months		49.54 (9.19)	50.50 (9.79)	50.76 (10.26)	—	–1.60 (–5.13 to 1.93)	–1.42 (–4.95 to 2.11)
4 months		49.08 (9.52)	50.25 (9.42)	50.62 (10.63)	—	–2.49 (–6.01 to 1.03)	–0.87 (–4.40 to 2.67)
6 months		48.60 (9.82)	50.04 (9.66)	50.57 (10.44)	—	–1.51 (–5.04 to 2.02)	0.11 (–3.42 to 3.65)
PSS^j^							
Baseline		16.97 (6.10)	17.25 (5.79)	16.28 (5.53)	7.46 (6)	–0.28 (–2.29 to 1.73)	–0.97 (–2.98 to 1.04)
2 months		16.21 (6.46)	16.93 (5.86)	15.53 (5.75)	—	–0.42 (–2.42 to 1.58)	–0.93 (–2.94 to 1.07)
4 months		15.50 (6.87)	16 (6.18)	14.99 (6.15)	—	–0.76 (–2.77 to 1.25)	–1.67 (–3.68 to 0.34)
6 months		14.54 (7.03)	15.56 (6.46)	14.42 (6.51)	—	–0.90 (–2.90 to 1.10)	–0.90 (–2.91 to 1.11)
WHO-5^k^							
Baseline		60.67 (19.77)	64.15 (19.63)	59.17 (20.99)	2.95 (6)	–3.49 (–10.42 to 3.45)	–1.78 (–8.71 to 5.15)
2 months		62.18 (20.64)	64.86 (19.77)	59.65 (22.38)	—	–1.11 (–8.04 to 5.82)	1.75 (–5.18 to 8.68)
4 months		62.54 (21.60)	64.96 (19.89)	60.21 (22.37)	—	–1.18 (–8.11 to 5.74)	3.75 (–3.18 to 10.68)
6 months		62.04 (22.67)	65.75 (19.89)	60.90 (22.47)	—	–0.31 (–7.24 to 6.62)	2.58 (–4.35 to 9.51)
Self-esteem^l^							
Baseline		30.54 (5.13)	30.57 (4.06)	30.51 (5.30)	5.60 (6)	–0.03 (–1.55 to 1.50)	0.03 (–1.69 to 1.75)
2 months		30.06 (5.27)	30.43 (4.15)	29.93 (5.31)	—	–0.38 (–1.94 to 1.19)	0.12 (–1.62 to 1.87)
4 months		31.82 (5.41)	29.89 (4.11)	29.74 (5.42)	—	1.93 (0.35-3.52)	2.08 (0.30-3.87)
6 months		31.44 (5.48)	29.64 (4.03)	29.46 (5.27)	—	1.81 (0.22-3.39)	1.99 (0.21-3.76)
Loneliness^m^							
Baseline		41 (9.71)	40.07 (9.56)	40.97 (9.68)	3.07 (6)	0.93 (–2.24 to 4.11)	0.03 (–3.17 to 3.22)
2 months		40.03 (9.93)	39.29 (9.78)	40.26 (10.07)	—	0.74 (–2.51 to 3.98)	–0.24 (–3.53 to 3.06)
4 months		34.49 (9.77)	38.25 (9.52)	39.58 (10.30)	—	–3.76 (–6.93 to -.58)	5.09 (–8.40 to –1.78)
6 months		33.55 (9.94)	37.35 (9.69)	38.57 (10.45)	—	–3.79 (–7.03 to –0.56)	–5.02 (–8.38 to –1.66)

a Adjusted means (SE) are estimated from linear mixed‐effects models with a random intercept for each participant, including fixed effects for group (1 = intervention, active control, 3=waitlist control), time (baseline, 2 months, 4 months, 6 months), and their interaction. Interaction effects were tested via likelihood‐ratio tests (chi-square (df)); reported chi-square and degrees of freedom refer to the comparison between the full model (with group × time) and the reduced model (without the interaction). Pairwise contrasts (intervention vs active control; intervention vs waitlist control) give mean differences and 2‐sided 95% CIs. Positive values indicate higher scores in the intervention group.

b LRT: likelihood ratio test.

c AC: active control.

d WLC: waitlist control.

e Not applicable.

f MET: total weekly metabolic equivalent minutes.

g GAD: Generalized Anxiety Disorder‐7.

h PHQ: Patient Health Questionnaire‐9.

i WEMWBS: Warwick–Edinburgh Mental Well‐being Scale.

j PSS: Perceived Stress Scale.

k WHO‐5: 5-item World Health Organization Well‐Being Index.

l Self‐Esteem: Rosenberg Self‐Esteem Scale.

m Loneliness: UCLA Loneliness Scale.

At 6 months, these benefits not only persisted but, in the case of BMI, became statistically significant. Although BMI differences were nonsignificant at 4 months, by 6 months the intervention group’s mean BMI was 0.47 kg/m² lower than that of the active control (95% CI –0.89 to –0.04; *P*=.04) and 0.54 kg/m² lower than that of the waitlist control (95% CI –0.85 to –0.01; *P*=.03). Weekly MET‐minutes remained elevated among intervention participants (129.45 MET-minutes greater than active control; 95% CI 53.28-205.61, *P*<.001; 152.87 MET greater than waitlist control; 95% CI 68.35-237.38, *P*<.001). Similarly, self‐esteem differences persisted: intervention scores were 1.81 points higher than active control (95% CI 0.22-3.39; *P*=.02) and 1.99 points higher than waitlist control (95% CI 0.21-3.76; *P*=.03). Loneliness remained lower in the intervention arm, with mean scores 3.79 points below active control (95% CI –7.03 to –0.56; *P*=.03) and 5.02 points below waitlist control (95% CI –8.38 to –1.66; *P*<.001; Figure 2; Table 2).

Besides these intervention effects, no differences in mental health, specifically depression, anxiety, and stress, were observed between conditions, and no adverse or unintended events occurred in any study arm. Participants’ evaluations of intervention quality did not differ between the intervention and control conditions, and follow‐up evaluations indicated high overall satisfaction rates among study participants.

## Discussion

### Principal Findings

In this RCT, a digitally delivered peer-support intervention yielded significant improvements in both physical and psychological outcomes among university students with preobesity. Compared with participants in the active control and waitlist control groups, those receiving digital peer support experienced a greater reduction in BMI over 6 months, accompanied by marked increases in weekly physical activity. Notably, the intervention also promoted significant enhancements in self-esteem and produced a meaningful decrease in loneliness scores. These convergent improvements demonstrate that a well-structured digital peer-support program can effectively address weight-related metrics while simultaneously bolstering mental well-being in a high-risk young adult population.

### Comparison with Prior Work

When contextualized within the broader literature, our results both corroborate and extend existing findings. Prior meta-analyses of peer-support interventions have reported modest reductions in weight and BMI, often limited to decreases on the order of 0.5‐1.0 kg and 0.1‐0.2 kg/m², respectively [48]. In contrast, the BMI improvement observed here exceeded those averages, suggesting that delivering peer support through a comprehensive digital platform may enhance engagement and efficacy. Similarly, earlier work by Yeo and colleagues found that digital peer-support programs led to higher levels of physical activity [24]. Our trial confirms that young adults can achieve and maintain substantial activity gains when supported by peers in a virtual setting. More striking is the contrast between our findings on psychosocial outcomes and those of many prior weight management trials. Whereas some research has found limited or no improvement in self-esteem or loneliness following peer support, often attributing these null effects to insufficient social cohesion or lack of personalized engagement [8], our intervention clearly produced robust psychological benefits. The structure of our digital program, which combined interactive workshops, ongoing peer dialogues, gamified challenges, and personalized goal-setting, likely fostered a sense of community and accountability that translated into higher self-esteem and reduced perceived social isolation.

### Potential Mechanisms

Several features of our study design and implementation may underlie these positive outcomes. First, we targeted college students with “preobesity,” a population at a critical inflection point that is often overlooked by both obesity-prevention initiatives and mental health programs [2]. By focusing on individuals with BMI between 25 and 29.9 kg/m², our intervention capitalized on a phase when behavior change is particularly feasible, as these students are frequently less entrenched in unhealthy habits and experience fewer physiological impediments compared to individuals already in the obese range [52]. Second, the digital nature of our peer-support platform enabled consistent, real-time interaction and social reinforcement, meeting students in their preferred communication channels. Daily micro-tasks, biweekly group challenges, and digital badges created an environment in which healthy behaviors became normalized and visible, thereby leveraging social comparison and accountability to strengthen motivation. Third, the inclusion of both an attention-matched active control and a waitlist control increased the rigor of our design. By demonstrating that the intervention’s benefits exceeded those of an equally engaging, wellness-focused program, we confirmed that the peer-support component itself was responsible for the superior outcomes, rather than the generic effects of participation or attention.

Behavioral and psychosocial theories provide mechanistic explanations for the observed effects. According to social cognitive theory, observing peers’ successes and receiving encouragement enhances self-efficacy, which in turn promotes adoption and maintenance of healthy behaviors [53]. In our study, participants saw fellow group members achieving step-count goals, sharing healthy meal ideas, and offering positive feedback, all of which contributed to a collective sense of possibility. As self-efficacy increased, participants likely felt more capable of meeting weekly activity targets and monitoring their weight, resulting in the higher MET scores and lower BMI values observed at 4 and 6 months. Concurrently, the online peer-support format fostered social connectedness and reduced feelings of isolation, with participants reporting a growing sense of belonging as they exchanged challenges and celebrated accomplishments in a respectful, nonjudgmental space [54]. Improvements in self-esteem likely stemmed from personal achievements, such as meeting fitness goals and from external validation like peer praise, thereby reinforcing a more positive self-concept [55]. Moreover, reductions in loneliness may have mitigated stress and negative affect, both of which are known barriers to sustained lifestyle change [54]. By alleviating emotional distress and enhancing social support simultaneously, the intervention created a synergistic environment in which mental well-being and physical health could rise together [56].

### Implications

Finally, the integration of physical, behavioral, and psychosocial measures in our outcome assessment underscores the value of holistic intervention approaches. In contrast to many programs that focus narrowly on either weight loss or mental health, our trial demonstrates that digital peer support can generate parallel benefits across multiple domains. This alignment is particularly important in the university setting, where academic pressures, social transitions, and evolving peer networks intersect to shape students’ health behaviors and psychological states. By addressing these factors in concert, providing evidence-based guidance on nutrition and exercise alongside structured opportunities for emotional support, the intervention enabled participants to tackle weight management without sacrificing mental health, and vice versa. Such a comprehensive strategy not only enhances immediate outcomes, such as lower BMI, higher activity levels, improved self-esteem, and reduced loneliness, but also establishes a foundation for sustained long-term well-being. Given the extensive reach and accessibility of digital platforms, our findings underscore the importance of integrating peer-supported programming as a foundational element of university health promotion strategies.

### Limitations

This study has several limitations. First, the use of self-reported weight and physical activity data may have introduced measurement error, given the well-established divergence between self-reported and objectively measured values. Second, relying on BMI as the sole physical outcome may not adequately capture changes in body composition, as BMI does not differentiate between fat and lean mass. Third, the study sample consisted exclusively of digitally literate, university students with preobesity, which may limit the generalizability of the findings to other populations, including individuals in different age groups, weight categories, or with limited digital access or engagement. Fourth, we observed no significant changes in some secondary outcomes. This null finding may be attributable to the relatively short follow-up period and limited sensitivity of the self-report instruments. Long-term follow-up is needed to determine whether improvements in BMI, physical activity, and mental health are sustained beyond the intervention and to evaluate any delayed effects on the incidence of obesity and mental health disorders. Fifth, we could not directly verify completion of some self-guided micro tasks at the individual level; future trials should incorporate objective sensing or device-based validation to confirm task completion without increasing participant burden.

### Conclusions

In this RCT among university students with preobesity, a digital peer-support intervention led to improvements in both physical and mental health outcomes compared to a control condition. Participants receiving the program attained a modest reduction in BMI and increased physical activity, while also reporting higher self-esteem and lower loneliness than controls. These findings highlight the promise of interventions that leverage peer support via digital platforms, an accessible and replicable approach to addressing emerging health risks in young adults. The intervention’s digital, peer-driven design enables broad dissemination across campus settings and beyond, engaging at-risk students who may not use traditional health services. Notably, this trial is among the first to target the preobesity stage with a holistic approach that concurrently addresses weight management and mental health. By intervening early with an integrated program, this approach prevents progression to obesity while mitigating psychological distress, illustrating a novel paradigm for young adult health promotion.

## Supplementary material

10.2196/78960 Checklist 1 CONSORT-eHEALTH checklist (V 1.6.1).

## Abbreviations

CONSORT: Consolidated Standards of Reporting Trials
GAD-7: Generalized Anxiety Disorder Scale 7
MET: metabolic equivalent of task
PHQ-9: Patient Health Questionnaire-9
PSS: Perceived Stress Scale
RCT: randomized controlled trial
SCS: Social Connectedness Scale
UCLA: University of California, Los Angeles Loneliness Scale
WEMWBS: Warwick-Edinburgh Mental Well-being Scale
WHO-5: 5-item World Health Organization Well-Being Index
